# Data on the effect of incorporation of nanoparticles and process characteristics of Ni–SiO_2_ coating on oil and gas steel

**DOI:** 10.1016/j.dib.2018.06.052

**Published:** 2018-06-21

**Authors:** Paul Apeye Lucky Anawe, Ojo Sunday Isaac Fayomi

**Affiliations:** a Department of Petroleum Engineering, Covenant University, P.M.B. 1023, Ota, Nigeria; b Department of Mechanical Engineering, Covenant University, P.M.B. 1023, Ota, Nigeria; c Department of Chemical, Metallurgical and Materials Engineering, Tshwane University of Technology, P.M.B. X680, Pretoria, South Africa

**Keywords:** Deposition time, Applied current density, Weight gain, Coating thickness

## Abstract

In this work, data on the effect of process parameter and particle concentration on the developed Ni–SiO_2_ produced via electrocodeposition were presented. The influence of current density from 0.6 to 1.0 A/dm^2^ and applied time difference from 10 to 25 min observed on the hardness characteristics and texture performance of the deposited alloy was checked. The weight gained and thickness of surface coverage was acquired and could be further used as a protoype for designing a ternary alloy coating system for oil and gas services.

**Specification Table**

**Table:** 

Subject area	*Corrosion Engineering*
More specific subject area	*Oil and Gas Refineries*
Type of data	*Table, image*
How data was acquired	The deposition took place in a designed electrodeposition sequence cell containing five steps according to the principle of electrolytic co-deposition route from pre treatment to post treatment. The coating thickness, weight gained, were measured using coating thickness gauge and weighing balance for the weight gain. The weight gain was obtained from the observed weight difference before and after coating.
Data format	Raw, Analyzed
Experimental factors	The admixed weight fraction of bath constituent was measured appropriately and electrolyte pH was obtained before the deposition was done and required data acquired.
Experimental features	The depositions were performed between 10 and 25 min at a stirring rate of 100 rpm at room temperature of 25 °C. The effect of coating difference on the properties and interfacial surface was acquired, at a current density interval between 0.6 and 1.0 A/dm^2^ for the coating duration. The framework of bath condition as it influences the coating thickness and weight gain was put into consideration.
Data source location	Surface Science Research Laboratory, Department of Mechanical Engineering, Covenant University, Ota Ogun State, Nigeria
Data accessibility	Data are available within this article

**Value of the data**

• The given data will show author in the field of corrosion engineering with interest in surface improvement the correlation between process parameter influence and incorporated particle on coating performance.
• The data obtained for the nickel-silicon dioxide electrolyte can be used as inference to determine the anomalous metal matrix co-deposition of ternary and quaternary alloy.
• The data can be used to examine the relationship between the process variable for instance (current density and time) as it affect the nature of coating stability.
• The data could be used at investigating the coating progression between the coating thickness, weight gain and the texture of the adsorbed deposits.
• The data obtained can be used in investigating the strengthening behaviour of particulate in an electrolyte relating to its mechanical characteristics.

## 1 Data

The data generated from the experiment are on variation of coating thickness, weight gain, at constant distance between the anode and cathode with depth of immersion. The depositions process was performed between 10 and 25 min at a stirring rate of 100 rpm at ambient temperature of 25 °C. The data acquired from spectrometer analysis of the mild steel is presented in Table 1. The coating depositions was run twice on two separate mild steel substrate from single electrolyte for all set of sample matrix to ascertain its repeatability in commercial form which is in par with study by Ref. [1]. The influence of coating thickness and weight gain were considered and each variable are acquire twice and the average taken as representative data for better precision. This repeatability in procedure was considered necessary because of the variation in current density and time of deposition to obtained the required data presented in (Table 2, Table 3, Table 4).

**Table 1:** Data showing the elemental chemical composition of mild steel.

**Element**	**% Content**	**Element**	**% Content**	**Element**	**% Content**
C	0.150	Mn	0.45	Si	0.18
P	0.01	Ni	0.007	Al	0.004
S	0.032	Nb	<0.005	Fe	Balance

**Table 2:** Data showing formulated design bath composition of Ni–SiO_2_.

**Composition**	**Mass concentration (g/L)**
NiSO_4_	100
SiO_2_	5–15
NaSO_4_	35
2 dimethylaminioethanol	5
Boric acid	10
Glycine	5
Thiourea	5
pH	4.6
Time	10–25 min
Current Density	0.6–1.0 A

**Table 3:** Data showing electrodeposition parameters and results for Ni–SiO_2_ plated mild steel.

**Sample numbers**	**Time (min)**	**Coating Thickness (μm)**	**Weight gain (g)**	**Current density (A/dm**^**2**^**)**
Ni–Si 1	10	105.2	0.022	0.6
Ni–Si 2	15	142.3	0.133	0.6
Ni–Si 3	20	150.2	0.146	0.6
Ni–Si 4	25	165.4	0.288	0.6
Ni–Si 5	10	120.2	0.082	0.7
Ni–Si 6	15	178.1	0.122	0.7
Ni–Si 7	20	165.5	0.179	0.7
Ni–Si 8	25	182.8	0.199	0.7
Ni–Si 9	10	132.1	0.090	0.8
Ni–Si 10	15	180.4	0.173	0.8
Ni–Si 11	20	170.1	0.192	0.8
Ni–Si 12	25	190.2	0.295	0.8
Ni–Si 13	10	142.8	0.358	0.9
Ni–Si 14	15	182.4	0.362	0.9
Ni–Si 15	20	172.6	0.381	0.9
Ni–Si 16	25	192.5	0.800	0.9
Ni–Si 17	10	162.2	0.365	1.0
Ni–Si 18	15	185.7	0.368	1.0
Ni–Si 19	20	198.3	0.391	1.0
Ni–Si 20	25	2000.5	0.895	1.0

**Table 4:** Data of Ni–Si physical observation trend.

**Sample nos.**	**Deposition time (min)**	**Deposition current *D* (A/dm**^**2**^**)**	**Physical plating effects**	**Weight of deposition (g)**	**Thickness of deposition (μm)**
Ni–Si 1	25	0.6	Diffused reflection	0.288	165.4
Ni–Si 2	25	0.7	Bright reflection	0.199	182.8
Ni–Si 3	25	0.8	Bright reflection	0.295	190.2
Ni–Si 4	25	0.9	Bright reflection	0.800	192.5
Ni–Si 5	25	1.0	Excellent reflection	0.896	200.5
(As-received)	25	–	–	–	–

## 2 Experimental design, materials and methods

Mild steel was commercially sourced and sectioned into (40 mm×20 mm×1 mm) sheet as cathode and 99.5% zinc plate of (30 mm×20 mm×1 mm) were prepared as anodes. The initial surface preparation was performed with finer grade of emery paper as described in our previous studies [2], [3], [4], [5]. The sample were properly cleaned with sodium carbonate, pickled and activated with 10% HCl at ambient temperature for 10 s then followed by instant rinsing in deionized water. The mild steel specimens were obtained from metal sample site in Nigeria. The chemical composition of the sectioned samples is shown in Table 1 as obtained from spectrometer analyzer. The electrolytic chemical bath of Ni–SiO_2_ fabricated alloy was performed in a single cell containing two nickel anode and single cathode electrodes as described by Refs. [6], [7], [8]. The distance between the anode and the cathode is 15 mm. Before the plating, All chemical used are analar grade and de-ionized water were used in all solution admixed. The bath was preheated at 25 °C. The processed parameter and bath composition admixed used for the different coating matrix is presented in Table 2. The choice of the deposition parameter is in line with the preliminary study from our previous work [9] (Table 5).

**Table 5:** Microhardness test result of Ni–Si deposited mild steel.

**Hardness depth (µm)**	**Ni–Si 1 (HVN)**	**Ni–Si 2 (HVN)**	**Ni–Si 3 (HVN)**	**Ni–Si 4 (HVN)**	**Ni-Si 5 (HVN)**	**As-received (HVN)**
0	92	95	97	99	105	40
20	139	142	162	175	182	41
40	138	144	169	179	186	38
60	130	132	164	180	193	36
80	132	136	168	192	200	35
100	136	144	170	193	205	36
120	138	146	172	194	210	42

**Fig. 1:**
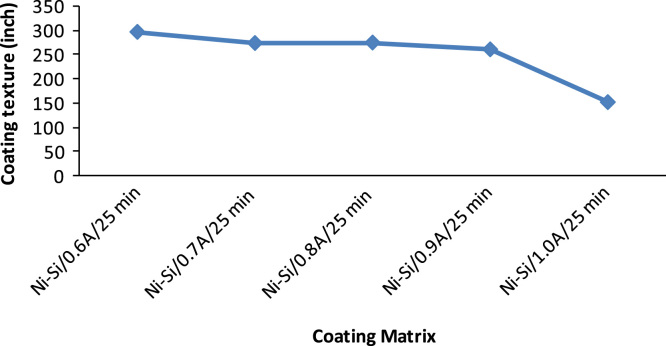
Variation of current density effect on coating texture.

The prepared zinc electrodes were connected to the rectifier at varying time of deposition and current density between 10–25 min and 0.6–1.0 A/cm^2^. The distance between the anode and the cathode with the immersion depth were kept constant as described by Ref. [3]. The fabricated were rinsed in distilled water and samples air-dried. Portion of the coating were sectioned for characterization (Fig. 1).

## 3 Conclusion

1. Nanostructure SiO_2_ particulates were used to produce Ni–SiO_2_ composite coating from sulphate bath.
2. There is a significant change in weight gain and coating thickness obtained from the variation of time of deposition and change in applied current density.
3. The hardness properties increases with increase in the process parameter.
4. There are excellent uniform distributions of coating texture from the obtained data.
