# Supplementary material for: Data on the effect of incorporation of nanoparticles and process characteristics of Ni–SiO2 coating on oil and gas steel
Source: Data Brief. 2018 Jun 21;19:1768–72. doi: 10.1016/j.dib.2018.06.052 (PMC6141766; doi:10.1016/j.dib.2018.06.052)
Supplement: Supplementary file 1 — Supplementary material [file mmc1.docx]

***COVER LETTER/CONFLICT OF INTEREST ATTESTATION***

*27th January, 2018*

*The Editor-in-Chief*

*Data in Brief*

***Subject:***  ***NO CONFLICT OF INTEREST***

*Dear Sir,*

*This serve to notify you that the manuscript is original of the authors work and there is no conflict of interest of any kind regarding the manuscript* **Data on the effect of incorporation of nanoparticles and process characteristics of Ni-SiO_2_ coating on oil and gas steel**

*Sincerely yours,*

*Prof. Anawe Paul*

*Department of Petroleum Engineering*

*Covenant University
Ota,*

*Nigeria*
